# MANF: A Novel Endoplasmic Reticulum Stress Response Protein—The Role in Neurological and Metabolic Disorders

**DOI:** 10.1155/2021/6467679

**Published:** 2021-10-28

**Authors:** Yang Yu, Dan-yang Liu, Xue-shen Chen, Ling Zhu, Li-hong Wan

**Affiliations:** ^1^NHC Key Laboratory of Chronobiology (Sichuan University), West China School of Basic Medical Sciences & Forensic Medicine, West China Second Hospital, Sichuan University, Chengdu, Sichuan 610041, China; ^2^Department of Neurosurgery, West China Hospital, Sichuan University, Chengdu, Sichuan 610041, China; ^3^Department of Pharmacology, West China School of Basic Medical Sciences & Forensic Medicine, Sichuan University, Chengdu, Sichuan 610041, China; ^4^Grade 2016, West China School of Basic Medical Sciences & Forensic Medicine, Sichuan University, Chengdu, Sichuan 610041, China

## Abstract

The mesencephalic astrocyte-derived neurotrophic factor (MANF), also named as arginine-rich protein (ARP) or arginine-rich mutated in early-stage tumors (ARMET), is a novel evolutionary conserved protein related to unfolded protein response. Growing evidence suggests that MANF critically involves in many ER stress-related diseases with a protective effect. Here, we review the function of MANF based on its structure in neurological and metabolic disorders and summarize its potential applications in disease diagnosis and therapies.

## 1. Introduction

Mesencephalic astrocyte-derived neurotrophic factor (MANF) is an evolutionary conserved endoplasmic reticulum stress- (ERS-) related protein, which was initially considered the neurotrophic factor (NTF) for the protective effect on midbrain dopamine neurons *in vitro* [1]. Classical NTF mainly regulates survival, growth, morphological plasticity, and synthesis of neurons, such as brain-derived neurotrophic factor (BDNF) and neurotrophin nerve growth factors (NGFs), whereas MANF as the noncanonical NTF extends the function of NTF, participating in the regulation of endoplasmic reticulum (ER) stress [2]. Compared with classic NTF, MANF possesses a distinct amino acid sequence [3], which contains the N-terminus being similar to saposin-like proteins [4] and the C-terminus being likely to SAF-A/B, Acinus, and PIAS (SAP) protein [5] indicating the different functions.

MANF is widely expressed in the brain of invertebrates and vertebrates, including *Caenorhabditis elegans*, *drosophila*, zebrafish, and rodents [6, 7], as well as peripheral nonneuronal tissues, such as liver, heart, kidney, salivary gland, testis, and pancreas [8]. In addition, in the central nervous system (CNS), MANF is abundantly expressed in several brain areas, such as the cerebral cortex, hippocampus (CA1-CA3 and dentate gyrus), substantia nigra (SN), and striatum [9].

Furthermore, emerging studies have demonstrated that MANF is mainly localized to the luminal endoplasmic reticulum (ER) as an ER chaperone protein [10, 11], mainly interacting with BiP [12–14] and Reticulon 1-C [15]. Further, following a range of ER stress, MANF expression significantly enhanced with increasing receptors activating transcription factor 6 (ATF6) and transcription factor X-box binding protein 1 (XBP1) [8, 16], both of which are ER stress markers. Similarly, the lack of endogenous MANF activated unfolded protein response (UPR) pathways and impaired neurite outgrowth and neuronal migration through UPR in MANF conditional deletion mice [17, 18].

**Figure 1:**
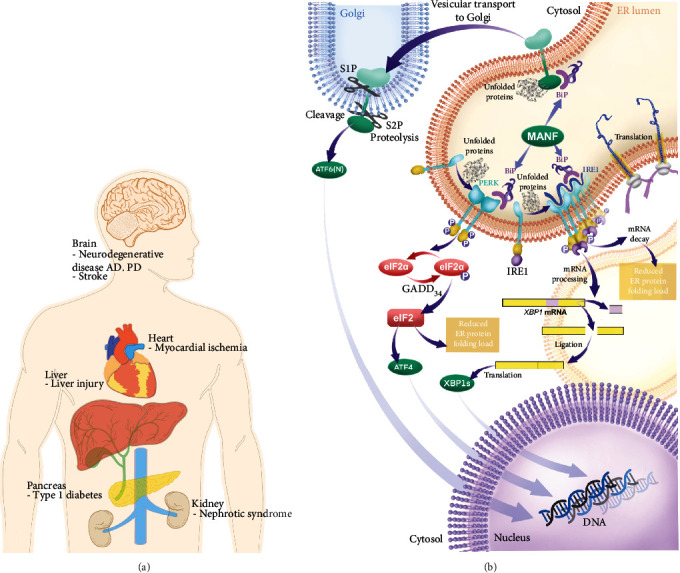
Hypothetic involvement of MANF in UPR pathway and related diseases. (a) MANF displayed expression variation and protective effects on neurological and metabolic diseases involving neurodegenerative diseases, myocardial ischemia, type I diabetes, and several tumors, which could be a potential prognostic marker or a therapeutic target. The circulating MANF variated in diabetes mellitus and PCOS [91, 92]. With bioinformatic analysis, increased expression of MANF was correlated with a poorer prognosis in cholangiocarcinoma and hepatocellular carcinoma [85, 86]. Moreover, MANF exhibited varying degrees of protective effects *in vitro* and *in vivo*. Overexpression or using recombinant MANF could promote dopaminergic neuron survival and improve behavior in neurodegenerative and stroke models. MANF overexpression ameliorated HepG2 cell steatosis and inhibited lipogenesis. Also, recombinant MANF protected cardiac myocytes from ischemia/reperfusion-mediated cell death. (b) When ER stress happens, ATF6, IRE2, and PERK separately activate, resulting in apoptosis and autophagy. MANF could interact with BiP, the endoplasmic reticulum chaperone, and finally promote cell survival. ATF6 would be transferred to the Golgi complex and be cleaved into two parts. Then, ATF6 acts as a transcription factor, resulting in the expression alteration of MANF, Xbp1, and CHOP. Meanwhile, IRE1 could be activated through phosphorylation and dimerization, resulting in upregulated expression of MANF and chaperones. Also, IRE1 enables JNK phosphorylation and upregulates the expression of NF-*κ*B, then inducing apoptosis and autophagy. The activated PERK effects with eIF2*α* leading to the translation of ATF4 and antioxidant response.

Meanwhile, MANF could prevent ER stress-mediated cell death in neurons [12] and other tissues, such as the liver, heart, and kidney [19–22]. The displayed protective function of MANF has the potential therapeutic value in several ER stress-related neurological and metabolic disorders, involving Parkinson's disease (PD) [23], Alzheimer's disease (AD) [24], stroke [19], and diabetes [25] (Figure 1(a)).

Despite success in preclinical studies, MANF has yet to fulfill its clinical potential facilitating diagnostics or therapies. Therefore, in the present review, we attempt to present the function of MANF in several neurological and metabolic disorders linked with its structure and illustrate the potential diagnostic or therapeutic application.

## 2. Molecular Structure of MANF

**Figure 2:**
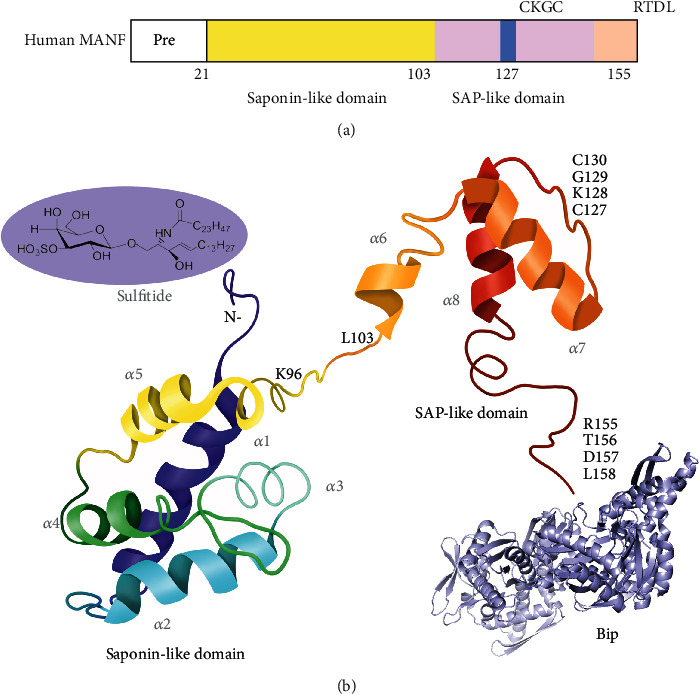
The structure of MANF. (a) The model structure of MANF. The Saposin-like domain was marked as green, and the SAP-like domain was marked as orange. (b) MANF is constructed with 158 amino acids and eight helices. The Saposin-like domain is located near the N-terminus, and the SAP-like domain stands on the C-terminus. CKGC in human MANF situates in 127^th^ to 130^th^ amino acids, being responsible for the neuroprotective effect. Besides, RTDL at the very end of MANF effects as a tag to locate the protein in the endoplasmic reticulum.

MANF is an evolutionarily conserved protein expressed in vertebrate and invertebrate animals, previously considered an arginine-rich protein [1, 26, 27]. Full-length human MANF protein, encoded by a 4.3 kb gene with four exons and located in 3p21.2, comprises 179 amino acids with 21 amino acids serving as a signal peptide (Figure 2(a)) [1]. The human *MANF* gene contains the arginine-rich sequence currently known as the part of 5′UTR without being translated. The secreted form of mature MANF contains 158 amino acids, and its molecular weight is 18 kDa [5]. Parkash et al. solved the crystal structures of full-length MANF at 2.8 Å resolution to X-ray crystallography [4]. The three-dimension structure of N-terminus (residues 1-95) is similar to saposin-like proteins (SAPLIPs), consisting of five *α*-helices and one 3_10_ helix and configured into a “closed leaf” structure through three disulfide bridges (Figure 2(b)) [4, 5]. This N-terminus facilitates MANF binding to sulfatide directly and exerts a protective effect [28], whereas the C-terminus (residues 104-158) is similar to SAF-A/B, Acinus, and PIAS (SAP) protein, containing a loose *α*-helix (*α*6) and two consecutive *α*-helices with helix-loop-helix DNA binding motif (*α*7 and *α*8) (Figure 2(b)) [1, 5]. And this SAP domain could connect with the nucleotide-binding domain of ADP-bound immunoglobulin heavy-chain-binding protein (BiP), helping MANF maintain protein folding homeostasis [14].

It is worth noting that the C-terminal domain of MANF is homologous to the Ku70 (an antiapoptotic protein by interacting with the proapoptotic Bcl-2-associated X protein (Bax)) SAP domain. Based on Ku70 and SAP's structural similarity, some researchers predict that MANF presumably acts as Ku70 [5]. However, no evidence shows the direct interaction between MANF and Bax [5, 29], but rather a connection via the Akt/MDM-2/p53 pathway [30, 31]. Also, SAP like-domain has been confirmed to facilitate MANF interacting with p65 and negatively regulate NF-*κ*B signaling under inflammation and ER stress [32].

Furthermore, some specific sequences in MANF C-terminus have been noticed, such as CXXC and RTDL motif. The CXXC motif is crucial for the neuroprotective activity of MANF. CKGC peptide could potently obstruct Fas-induced apoptosis and neutralize the reactive oxygen species generated by the cysteine residues [33]. Also, the MANF with CKGC mutation did not show the protective function in a cerebral ischemia rat model nor the survival-promoting function in cultured neurons [29].

As for the intracellular MANF, the RTDL triggers protein restoration from the Golgi complex to the ER. And the RTDL is located at the end of the C-terminus with Lys-Asp-Glu-Leu (KDEL) and recognized by KDEL receptors in the ER membrane [34]. When the RTDL was removed in cultured cells, the MANF would concentrate on the Golgi complex and lose its growth promotion effect [34, 35].

## 3. The Interaction of MANF with ER Stress-Related Protein

UPR as the adaptive reaction that happened under ER stress is the onset through BiP circumstantial interaction with PKR-like ER kinase (PERK), activating transcription factor 6 (ATF6), and inositol-requiring enzyme-1 (IRE1) [36]. BiP enables IRE1 and PERK to keep inactive and bind to ER lumenal domains in unstressed cells, while these ER stress-related proteins, IRE1, PERK, and ATF6, are released from BiP under ER stress, activating corresponding pathways [37, 38]. Specifically, PERK is activated by transautophosphorylation and phosphorylates eukaryotic initiation factor-2*α* (eIF2*α*), upgrading activating transcription factor 4 (ATF4) to attenuate protein translation via downstream transcription factor CHOP [39, 40]. The banding of ATF6 and BiP is disrupted under ER stress leading to ATF6 transformation to the Golgi complex. After twice cleavage, ATF6 transfers to the nucleus and activates URP target genes [41]. IRE1 is allosterically activated by transautophosphorylation and mediates X-box binding protein-1 (XBP1) cleavage with an endoribonuclease, consequently regulating URP-related genes [42].

Previous studies displayed an association between MANF and other ER stress-related proteins (Figure 1(b)). The intracellular MANF level could be enhanced by overexpression of BiP, the critical regulator in ER stress [43]. And MANF secretion is affected by its binding to BiP with calcium dependence [35]. Currently, it was demonstrated that secreted MANF could bind to Neuroplastin (NTPN), a cell surface receptor, and modulate inflammatory response through the NF-*κ*B pathway [44]. Moreover, MANF restrained ADP release from BiP and consequently stabilized the BiP-client complex [14], while the interaction of MANF and BiP is independent of its cytoprotective effects on the neuron [13]. Concretely, MANF R133E, E153A, and R133E E53A mutants hindered the interaction of BiP and MANF, but all these mutants did not reduce superior cervical ganglion neuron survival under tunicamycin. Also, MANF expression could be upregulated by an ER stress response element- (ERSE-) II in the MANF promoter, which is recognized by ATF6 and XBP1 [16, 45].

## 4. Functions of MANF in Neurological Disorders

### 4.1. Neurodegenerative Disorders

Neurodegenerative disorders are aging-related, progressive, and clinical incurable disorders characterized as brain dysfunction and neuronal death, such as Parkinson's disease (PD) and Alzheimer's disease (AD). It is well known that the typical clinical symptoms of PD include bradykinesia, rigidity, and tremors due to the lack of dopamine synthesis in the substantia nigra (SN) caused by dopaminergic neuron death [46]. Since Petrova et al. reveal the selective neuroprotective effect of MANF on dopaminergic neurons [1, 47], growing researches focus on elucidating MANF's role and mechanism in PD.

Recently, a clinical study from Finland showed that circulating MANF concentrations of PD patients were significantly increased, implying the potential role of MANF as a clinical marker for early-stage PD [48]. Moreover, emerging evidence indicates that MANF protects against dopaminergic neuron degeneration [49], especially preventing 6-OHDA-induced degeneration of dopaminergic nerves [23]. Also, MANF improves the mitochondrial function and alleviates the oxidative stress in a MPTP/MPP+-induced PD model [50]. Considering the regulation of ER stress on MANF expression and secretion, the molecular mechanism of MANF on protecting DA neurons focused on ER stress-related mitochondrial dysfunction and cell death, including apoptosis and autophagy [23]. In the *α*-synuclein induced *Caenorhabditis elegans* PD model, MANF lost its beneficial effect after silencing ER stress-related genes (BiP, UPR sensors IRE1, PERK, and ATF6) or autophagy-related genes (for example, AMPK/mTOR) [7]. On the contrary, MANF treatment presented a protective role in 6-OHDA-induced neurotoxicity through inhibiting AMPK/mTOR-mediated autophagy [51]. Similarly, in SH-SY5Y cells, both 6-OHDA and *α*-synuclein induced significantly neuronal apoptosis, which was reversed by MANF treatment through the upregulation of ER stress-related protein BiP [52, 53]. These studies all suggest that MANF may possess a neuroprotective role in regulating ER stress-related dopaminergic neuron death in PD. Notably, exogenous MANF alleviated 6-OHDA-induced cell damage and oxidative stress in SH-SY5Y cells via activating the Nrf-2 involved PI3K/Akt/GSK3*β* pathway [54] and ROS-AMPK/mTOR pathway [51]. The difference between overexpression and exogenous MANF might be attributed to the exogenous MANF not entering the cells [5].

Alzheimer's disease (AD) is another common neurodegenerative disorder caused by extensive neuroinflammation and massive neuron loss [55]. Recent studies in various inflammation models show that MANF protects against inflammatory response by reducing ER stress-related proteins [56] and negatively regulating the NF-*κ*B pathway [32, 57]. Importantly, A*β*_1–42_ significantly induced MANF expression, accompanied by ER stress-mediated neuron apoptosis *in vivo* and *in vitro*. Moreover, secreted MANF demonstrated the neuroprotective role in A*β* toxicity via attenuating ER stress, proven through knockdown of endogenous MANF and overexpression of MANF *in vitro* [24].

### 4.2. Stroke

In 2008, MANF mRNA and protein were found to increase transiently after cerebral cortex ischemia in a rodent model [8]. Subsequently, a series of evidence indicated that pretreatment with recombinant MANF or AAV vector containing human MANF cDNA (AAV-MANF) could significantly reduce the infarction area, alleviate the cognitive impairment, and improve the prognosis in a rat model [12, 58–60], indicating the protection of MANF on ischemic brain injury. Similar to neurodegenerative disorders, suppression of cell necrosis/apoptosis in the cerebral cortex via downregulation of BiP, p-IRE1, and XBP1s contributed to the neuroprotection effect of MANF on ischemic brain injury [56, 59]. Moreover, Shen et al. found ischemia-induced MANF expression of the activated microglia via regulating ER stress [61], which broadens our understanding of the mechanisms of MANF on the ischemia-induced neural injury. Besides, MANF was also demonstrated to promote angiogenesis via activation of the VEGF/VEGFR2 pathway [62] and promote migration of neural progenitor cells toward the infarct boundary by activating STAT3 and ERK1/2 [63].

## 5. Functions of MANF in Metabolic Disorders

### 5.1. Myocardial Ischemia

In 2008, Tadimalla et al. found that MANF expression increased in the border zone and infarct zone in a mouse myocardial infarction model colocalized with BiP, which was increased in an ATF6-dependent manner [64]. MANF knockdown mice demonstrated a significant increase in myocardial damage and decreased cardiac function during simulated ischemia/reperfusion [22]. In contrast, recombinant MANF protected cardiac myocytes from ischemia/reperfusion-mediated cell death and decreased myocardial damage to the myocardial infarction model by reducing ER protein misfolding [35]. These results may suggest that MANF has application prospects for myocardial ischemia.

### 5.2. Liver Disease

MANF is involved in multiple hepatic metabolic diseases as a secreted protein, including virus- or alcohol-induced liver injury and cancer, through interacting with XBP1 [65–69]. In detail, the transcription of MANF is enhanced by XBP1 activation [69]. MANF also regulates its expression through binding to XBP1s. Moreover, the role of MANF from different sources in liver disease is different. Immune cell-derived MANF protects against liver inflammation and fibrosis, whereas hepatocyte-derived MANF prevents hepatosteatosis [68].

The protective effect of MANF on liver injury was first reported in nonalcoholic fatty liver disease [70]. MANF expression was increased early and gradually decreased afterward under high free fatty acid stimulation in HepG2 cells. The loss of MANF accelerated lipogenesis and aggravated HepG2 cell steatosis. At the same time, MANF overexpression inhibited lipogenesis and rescued HepG2 cell steatosis from free fatty acid treatment, indicating that MANF may be a potential therapeutic target in hepatic steatosis processes [70]. Meanwhile, MANF could alleviate diet-induced obesity through adipose browning with the effects of the p38 MAPK pathway [71]. Also, MANF was found to be upregulated and inhibit liver cancer and inflammation via SUMOylation-related suppression of the NF-*κ*B/Snail signaling pathway and epithelial-mesenchymal transition (EMT) [67]. SUMO1 overexpression increased MANF nuclear import in mouse HCC induced by oxygen and glucose deprivation (OGD) and strengthened the interaction between MANF and p65. SUMOylation of p65 recruits MANF to form a repressor complex to shut down NF-*κ*B signaling, leading to the downstream genes (Snail1 and TNF-*α*) of the NF-*κ*B signal pathway being inhibited. Subsequently, hepatocyte EMT and HCC were suppressed [67].

### 5.3. Kidney and Pancreatic Diseases

MANF can be detected in the urine of podocyte endoplasmic reticulum stress models, including passive Hyman nephritis and puromycin mononucleotide nephropathy [72]. Kim et al. reported for the first time that MANF potentially served as a urinary ER stress biomarker in ER stress-mediated kidney disease [72, 73]. In the podocyte ER stress-induced hereditary nephrotic syndrome (NS) mouse model, MANF was induced and secreted by ER-stressed podocytes at the early stage of proteinuria. Most importantly, urinary MANF excretion concurrent with podocyte or tubular cell ER stress preceded clinical or histologic manifestations of the corresponding disease [74, 75], indicating that MANF served as urine diagnostics, a prognostic biomarker in ER stress-related kidney diseases. Furthermore, MANF was shown as an ER calcium channel stabilizer in the NS mouse model to inhibit podocyte injury via fixing leaky type 2 Ryanodine receptor (RyR2) [21]. Plus, MANF could inhibit inflammation and renal M1 macrophage in the acute kidney injury model [76].

MANF is also strongly expressed in pancreatic exocrine acinar cells and endocrine islet *β*-cells [66]. Insulin-producing *β*-cells lacking MANF develop severe chronic ER stress leading to decreased *β*-cell proliferation and cell deaths, eventually leading to type 1 diabetes (T1D) [66, 77, 78]. Recently, Montaser et al. exhibited that MANF deficiency would cause dysfunction of human *β*-cell and increased ER stress [79]. On the contrary, delivery of MANF to the mouse pancreas via a viral vector induced regeneration of *β*-cells in vivo in a T1D model and, notably, increased proliferation of both mouse and human *β*-cells *in vitro* [66]. Besides, MANF protects human islet cells by inhibiting the NF-*κ*B signaling pathway and improving ER stress [80–82]. Thus, MANF might be a potential therapeutic target for alleviating ER stress, rescuing *β*-cells, and inducing *β*-cell regeneration in diabetes.

## 6. Diagnostic and Therapeutic Perspectives for MANF

MANF is currently considered a potential diagnostic or prognostic marker and treatment target due to its protective role in multiple ER stress-related diseases. Human *MANF* variation of multiple AGG repeats has been confirmed in several tumors involving the lung, breast, prostate, esophageal, and pancreatic cancer [83, 84]. Moreover, MANF overexpression in hepatic carcinoma is associated with a high risk of occurrence [85]. Analogously, patients with MANF overexpression had shorter overall survival and progress-free survival in cholangiocarcinoma [86]. Except for tumors, MANF could be the diagnostic or prognostic factor in some metabolic diseases. It was found that circulating MANF upgraded in newly diagnosed type 2 diabetes mellitus than normal glucose tolerance [87]. Interestingly, circulating MANF significantly decreased in polycystic ovary syndrome (PCOS) patients with insulin resistance. Compared with the previously mentioned study, the diverse tendency is probably caused by the different periods of the disease. MANF degradation increased following prolonged ER stress but expressively upgraded in acute ER stress [10, 80]. Compared with relatively abundant clinical study about metabolic disorders, MANF-related clinical study about neurologic disorders seems less concerned, though the preclinical studies exhibited potential therapeutical values in PD and stroke. The terms MANF, ARP, and ARMET were used to search relevant clinical studies on the Chinese Clinical Trial Registry and Clinical Trials.gov with results displayed in Table 1.

Existing studies primarily focused on the role of recombinant human MANF (rhMANF) supplement. Potential drugs and regulatory effects on MANF were reported recently, such as liraglutide [82] and valproic acid [88]. All potential drugs targeting MANF retrieved by PubMed are shown in Table 2. Continual efforts are required to explore more drugs targeting MANF in the future.

## 7. Research Prospects

Currently, the structure and function of MANF have been partially demonstrated. MANF displayed a protective effect on central nervous system diseases and played an important role in several metabolic diseases. Moreover, we could expect progress in several further research directions. First, the mechanism of MANF was relatively unclear. Currently, merely one study revealed the interaction of secreted MANF and its receptor, NTPN, which modulates inflammation through the NF-*κ*B pathway [44]. And there is no other evidence to show how the secreted MANF functions. Further, current researches mainly focused on the mechanism of MANF in ER stress. Other potential mechanisms were seldom elaborated. Besides, another future research priority is to accelerate the transformation and application of MANF. The locally administrated recombinant MANF has exhibited an excellent neuroprotective effect, improving behavior in several nervous system disease models. However, there is no evidence of pharmacodynamics and pharmacokinetics of the recombinant MANF. The recombinant protein is commonly unstable and easily devitalized. Therefore, it is urgent to make modifications on MANF so that we can optimize the performance on pharmacodynamics and pharmacokinetics and finally realize the clinical application.

## 8. Conclusions

Here, we summarized the structure of MANF and the significant evidence highlighting the important functions of MANF in metabolic and neurological disorders. MANF might be a potential therapeutic target, and screening of drugs regulating MANF will facilitate the treatment of various diseases related to ER stress.

## Figures and Tables

**Table 1:** Registered clinical studies related to MANF.

Title	Study start date	Disease	Study type
Tracking neurodegeneration in early Wolfram syndrome (TRACK)	April 2012	Diabetes insipidus, diabetes mellitus, Wolfram syndrome	Prospective observation
Effect and mechanism of MANF on hepatocellular cancer	Aug. 2018	Hepatocellular cancer	Retrospective observation
Effect of MANF in pulmonary inflammation and inflammation-associated brain dysfunction	Aug. 2018	Pneumonia, cognitive dysfunction	Retrospective observation

**Table 2:** Potential drugs targeting MANF.

Drug	Subject	Target	Mechanism
Valproic acid	Rat	Brain	mRNA and protein expression of MANF increased
Piperine [89]	Mouse and PC12 cell	Brain	Antagonize ER stress by activating MANF expression
Paroxetine [90]	Primary astrocytes		Increasing mRNA expression of MANF
Metformin [91]	Human	Serum	Elevating serum MANF levels in PCOS women
Liraglutide [82]	Human	Serum	Upregulating MANF
